# Effect of Pharmacist-Led Interventions on Physicians’ Prescribing for Pediatric Outpatients

**DOI:** 10.3390/healthcare10040751

**Published:** 2022-04-18

**Authors:** Kien Trung Nguyen, Vy Tran Thanh Le, Thao Huong Nguyen, Suol Thanh Pham, Phuong Minh Nguyen, Katja Taxis, Mai Tuyet Vi, Thang Nguyen, Hung Do Tran

**Affiliations:** 1 Faculty of Medicine, Can Tho University of Medicine and Pharmacy, Can Tho City 900000, Vietnam; ntkien@ctump.edu.vn (K.T.N.); nmphuong@ctump.edu.vn (P.M.N.); 2 Department of Pharmacy, Can Tho Children’s Hospital, Can Tho City 900000, Vietnam; thanhvy121192@gmail.com; 3 Department of Clinical Pharmacy, School of Pharmacy, University of Medicine and Pharmacy at Ho Chi Minh City, Ho Chi Minh City 700000, Vietnam; huongthao0508@gmail.com; 4 Department of Pharmacology and Clinical Pharmacy, Can Tho University of Medicine and Pharmacy, Can Tho City 900000, Vietnam; ptsuol@ctump.edu.vn (S.T.P.); maivivi127@gmail.com (M.T.V.); nthang@ctump.edu.vn (T.N.); 5 Groningen Research Institute of Pharmacy, University of Groningen, 9713 AV Groningen, The Netherlands; k.taxis@rug.nl; 6 Faculty of Nursing and Medical Technology, Can Tho University of Medicine and Pharmacy, Can Tho City 900000, Vietnam

**Keywords:** drug-related problems, outpatients, pediatrics, prescribing, Vietnam

## Abstract

Background: Children are at high risk of drug-related problems, increased risk of treatment failures, and high treatment costs. We aimed to evaluate the effect of pharmacist-led interventions on physicians’ prescribing for pediatric outpatients. Methods: A prospective study with pre- and post-intervention measurement assessment was conducted to collect pediatric outpatients’ prescriptions during the pre-intervention period (January 2020) and post-intervention (August 2020) at a children’s hospital in Vietnam. Drug-related problems were identified and categorized according to Pharmaceutical Care Network Europe (PCNE), version 9.1. The intervention program was developed based on the results of pre-intervention observations. After the intervention, prescriptions were evaluated. Statistical tests were used to compare the proportions of drug-related problems before and after the intervention and to identify factors related to drug-related problems. Results: There were 2788 out of 4218 (66.1%) prescriptions with at least one drug-related problem before the intervention. Of these drug-related problems, the most common was inappropriate timing of administration and incorrect dosage (36.1% and 35.6%, respectively). After the intervention, the percentage of prescriptions with at least one drug-related problem was 45.5% (*p* < 0.001). Most of the drug-related problem types decreased significantly (*p* < 0.05). The binary logistic regression analysis results showed that in addition to pharmacists’ intervention, patients’ gender, primary disease, comorbidity status, and the total number of drugs prescribed were also factors related to drug-related problems. Conclusions: Drug-related problems in pediatric outpatients were quite common. Pharmacists’ intervention helped to improve the prevalence and types of drug-related problems.

## 1. Introduction

Inappropriate drug use in disease treatment and health enhancement can lead to drug-related problems (DRPs), defined as events or circumstances involving drug therapy that actually or potentially interfere with desired health outcomes [1]. DRP is a broad term that includes other problems such as medication errors (ME), adverse drug events (ADE), and adverse drug reactions (ADR) [2]. There are currently many classification systems for DRPs, such as APS Doc [3], Cipolle et al. [4], DOCUMENT [5], PCNE [1], and SFPC [6]. Each classification system is divided into several problem groups and subgroups. Depending on healthcare settings, pharmacists often use existing or based classification systems to develop and adapt the appropriate one to identify and classify DRPs [1,3,7].

The pediatric population is at higher risk of being affected by DRPs, as “Children are not just “little adults” in that they exhibit essential individual variation in organ development, weight, and body surface area, which makes drug therapy more complicated than in adults [8]. Initially, more detailed information on clinical trials and the safety of drugs in pediatric patients is not yet available, especially for children under two years of age. Thus, physicians prescribe for pediatric patients based on off-label indications [9,10]. Furthermore, a lack of appropriate dosage form and weight-based dose calculation are risk factors leading to DRPs in children [11,12]. The occurrence of DRPs accounted for a high percentage of pediatric inpatients and even outpatient prescriptions, reaching from 52.9% to 87.7% [8,13,14,15]. DRPs prevalently occur during the process of prescribing medication, including drug selection, dosage, and route of administration, especially dosing-related issues, which ranked the highest in frequency [14,16,17]. Patients’ disease status can worsen because of DRPs, prolonging the hospital stay or re-hospitalization, which leads to increased treatment costs. Therefore, it is necessary to identify DRPs and provide solutions to reduce them to improve treatment effectiveness for pediatric patients.

Marcela Urtasuna et al. reported that implementing electronic prescription systems in the management of prescriptions resulted in a decrease of 30% in medication errors in pediatric patients [18]. In addition, there is considerable evidence supporting the role of clinical pharmacists in detecting and minimizing the DRPs occurrence, particularly DRPs in relation to the pediatric prescribing process [19,20,21]. In Vietnam, many healthcare facilities have some problems with inappropriate prescribing, including the overuse of antibiotics and vitamins, over-prescribing of medication, and inadequate prescription drugs instructions. These issues cause the obstacle to clinical pharmacists making interventions in prescribing, especially for pediatric outpatients, since children’s use of medication is administered and tracked by parents at home. Therefore, we aimed to evaluate the effect of pharmacist-led interventions on DRPs related to prescribing for pediatric outpatients.

## 2. Materials and Methods

### 2.1. Study Design and Population

We conducted a prospective study at each stage with pre-intervention (January 2020) and post-intervention (August 2020) measurement assessments at an outpatient clinic of a children’s hospital in Can Tho City, Vietnam. We collected all prescriptions of pediatric outpatients from 0 to 16 years old between January and October 2020. These prescriptions were from physicians who had a fixed outpatient prescribing schedule at the hospital. We excluded prescriptions by physicians preparing to attend long-term training classes or who would be on maternity leave or absent for other reasons at least nine months after the study started. Moreover, we excluded prescriptions given to the same pediatric patient at follow-up examinations during each pre- and post-intervention period. These prescriptions lacked sufficient patient information regarding patient age and weight.

### 2.2. DRPs Measurement

Pre-intervention prescriptions were evaluated, and DRPs were identified by clinical pharmacists based on prescription and medication use references such as the instruction manuals, Pediatric Treatment Regimen (outpatient part), National Pharmacopoeia of Vietnam, and British National Formulary for Children [22,23,24,25]. Drug–drug interactions were identified using Lexicomp^®^ (Wolters Kluwer Health, Hudson, NY, USA) and Micromedex^®^ software (IBM Watson Health, NY, USA). Clinical pharmacists considered any nonconformity to one of the above sources to be a DRP and classified it according to the PCNE 9.1 system [1]. Based on the identified types of DRPs, clinical pharmacists developed appropriate intervention programs to provide physicians with more information about drugs and DRPs. Post-intervention prescriptions were reevaluated to determine the effectiveness of pharmacists’ intervention in reducing the incidence of prescription-related DRPs. The primary evaluation criterion was the percentage of prescriptions with at least one DRP, and sub-criteria involved the percentage of prescriptions related to each type of DRP.

### 2.3. Pharmacist-Led Interventions

The intervention content included the report on DRP cases related to outpatient prescriptions, the impact of DRPs on effectiveness, safety, and cost, and the active ingredients identified in each DRP group. Pharmacists intervened in DRPs for three weeks in the following ways: preparing content and reporting DRPs during a hospital briefing session in the first week, providing information on DRPs to the physicians in the second week, and repetition of the information in the third week. Moreover, during the intervention stage, the clinical pharmacists were also in charge of answering questions and providing any additional information required by the physicians. During the intervention, all information provided to physicians was taken from the database sources used to determine DRPs. A detailed description of the content and implementation of each step of the pharmacists’ intervention program is presented in Table 1.

### 2.4. Data Management and Analysis

Data were analyzed using Microsoft Excel 2019 (Microsoft, Redmond, WA, USA) and IBM SPSS statistics 26.0 software (IBM, Armonk, NY, USA). Qualitative variables (patient characteristics, DRPs types) were expressed in frequency and percentage. We compared the differences in patient characteristics and DRPs in the prescriptions, and pre- and post-intervention evaluation criteria, using statistical tests with 95% confidence. The difference was considered statistically significant when *p* < 0.05. We used a chi-square test to compare two ratios; when more than 20% of the comparison table cells had an expected value of <5, the Fisher test was used. To compare two mean values (average number of DRPs per prescription) for two independent samples (pre- and post-intervention), we used the Independent-Sample *t*-test for quantitative variables with normal distribution and the Mann–Whitney U test for quantitative variables without normal distribution.

In addition to pharmacists’ intervention, patient characteristics might be risk factors for DRPs. Therefore, to determine the impact of pharmacists’ intervention on the occurrence of DRPs, we used a multivariate logistic regression model, the variable Enter method. The dependent variable was DRPs (prescription with DRPs or not DRPs). Independent variables were pharmacists’ intervention (pre-intervention, post-intervention), age group (≤2 years old, >2 to ≤6 years old, and >6 years old), gender (male, female), primary disease (respiratory system, other diseases), comorbidities (yes, no), and total drugs in prescription (<5 drugs, ≥5 drugs). A *p*-value <0.05 was statistically significant.

### 2.5. Ethics Approval

The study was approved by the Ethics Committee in Biomedical Research of Ho Chi Minh City University of Medicine and Pharmacy, with code 346, issued on 26 May 2020. The collected information was kept confidential and used only for research purposes.

## 3. Results

### 3.1. Characteristics of the Study Population

We collected 4218 pre-intervention prescriptions and 4182 post-intervention prescriptions. Patients’ age and sex distribution in the prescriptions did not significantly differ between pre- and post-intervention (*p* > 0.05). However, there were significant differences in the primary disease characteristics, comorbidity status, and the total number of drugs in each prescription (*p* < 0.001). Because the differences in these characteristics could affect the post-intervention rates of DRPs, it was necessary to include these variables in binary logistic regression analysis to consider their relevance to the intervention results. Patient characteristics in pre- and post-intervention prescriptions are presented in Table 2.

### 3.2. DRPs in Prescriptions Pre- and Post-Intervention

The percentage of prescriptions with at least 1 DRP before the intervention was relatively high (66.1%), but after the intervention, the figure showed a significant decrease to 45.5% (*p* < 0.001). Similarly, the average number of DRPs per prescription and the proportion of each type of DRP significantly reduced after the intervention (*p* < 0.001), except for drug–drug interactions. However, after the pharmacists’ intervention, the proportion of dose timing relative to meals was still relatively high (32.6%) compared with the rest of the DRP groups such as dose selection (15.6%), dosage form (3.3%), and drug choice (2.8%). DRPs in pre- and post-intervention prescriptions are presented in Table 3.

### 3.3. Pharmacist Intervention Efficacy and Factors Related to DRPs

Binary logistic regression analysis indicated that post-intervention prescriptions were less likely to lead to DRPs than pre-intervention prescriptions (OR = 0.478; 95% CI 0.436–0.524); these results were statistically significant (*p* < 0.001). Moreover, patient gender, primary disease, comorbidity status, and the total number of drugs in prescription were also factors associated with the occurrence of DRPs. Factors related to DRPs are presented in Table 4.

## 4. Discussion

### 4.1. Characteristics of the Study Population

The difference in patient age and gender characteristics in the prescriptions before and after the intervention was not statistically significant (*p* > 0.05). Children ≤ 6 years old accounted for most cases, at nearly 80.0%. Males accounted for a higher rate than females, at about 1.2%, similar to previous studies on DRPs in children [8,14,15,17,19,20]. The primary diseases, comorbidity status, and the total number of drugs in the prescription differed significantly between the two phases (*p* < 0.001). In the post-intervention period, respiratory system diseases still accounted for the highest proportion but had decreased by about 20.0% compared to the pre-intervention period (78.0% and 58.0%, respectively). Some illnesses in children often have seasonal peaks: respiratory diseases usually occur more often in the last months of the year, and gastrointestinal diseases occur more often in the summer. Because the sampling times of the two study phases differed, there were differences in the primary diseases diagnosed. The above reasons also explain the differences in the features of comorbidity status between the two stages. Furthermore, in the post-intervention period, the number of prescriptions with ≤5 drugs was lower than in the pre-intervention period; this was explained by the differences in primary diseases leading to the different total of drugs prescribed or by the extra caution of physicians in prescribing unnecessary drugs following the pharmacists’ intervention. Therefore, to consider whether the pre- and post-intervention differences in the above factors affect the intervention’s effectiveness, we included these variables in binary logistic regression to examine their relevance to the appearance of DRPs in outpatient prescriptions.

### 4.2. DRPs in Prescriptions Pre- and Post-Intervention

After the intervention, we found that the proportion of prescriptions with at least 1 DRP decreased from 66.1% to 44.5%, and the mean number of DRPs per prescription also decreased from 0.93 to 0.56 DRPs (*p* < 0.001). The above results indicate that the pharmacists’ intervention helped physicians be more cautious in prescribing. The updated information provided by pharmacists or in other documents minimized the occurrence of DRPs related to prescriptions.

Pharmacists’ interventions on DRPs in several other studies showed similar effects. The intervention study of R.F. Budiastuti (2019) in pediatric patients with acute lymphoblastic leukemia reduced the number of DRPs from 177 pre-intervention to 10 post-intervention. In this study, the pharmacist intervened by reporting and discussing DRPs face-to-face with the physicians, nurses, and dietitians to adjust treatment therapies [20]. Previously, V.A. Sagita (2018) also similarly intervened regarding DRPs in cardiovascular disease patients ≥ 35 years old and showed a reduced number of both DRPs and types of DRPs [26].

Some other studies evaluated the effectiveness of interventions based on the proportion of problems accepted by the physician. After the pharmacists’ intervention, physicians agreed to adjust their prescriptions at 73.5% and 93.0% [13,14,27]. Due to different evaluation methods, it was difficult to compare the results of our study with those of others regarding the intervention’s effectiveness on DRPs. However, the above results indicate that the clinical pharmacists’ intervention reduced prescriptions related to DRPs.

#### 4.2.1. Drug Choice

The proportion of prescriptions involving DRPs related to drug selection decreased from 7.0% to 2.8% after the intervention (*p* < 0.001). These cases indicated that the prescription of drugs not suited to the diagnosis, and those not ideal for patients, decreased significantly after the intervention. However, the pre- and post-intervention rates of these DRPs were lower than in previous studies on inpatients and outpatients [8,15,28]. Over-prescribing and prescribing drugs with contraindications that do not increase the therapeutic benefit of the disease can lead to adverse drug reactions (ADR) in patients and unnecessary treatment costs. Therefore, the pharmacists’ intervention on DRPs related to drug selection was quite favorable and received physicians’ approval.

#### 4.2.2. Dosage Form

After the intervention, this rate decreased significantly to 3.3% (*p* < 0.001). Unlike adults, medicines for children vary in dosage forms, such as powder for oral suspension, oral syrup, oral solution, and oral tablets. Each form would be suitable for different age groups. Thus, we recognized the importance of intervention and drug information by clinical pharmacists for the prescribing physicians. This might help pediatric caregivers select the correct drugs and give appropriate doses to the children in their care. Moreover, the drugs would be better absorbed, providing the best treatment effect.

#### 4.2.3. Dose Selection

Prescriptions with inappropriate doses decreased significantly, from 35.5% before the intervention to 15.6% after the intervention (*p* < 0.001); both high doses and low doses decreased (*p* < 0.001), indicating the effectiveness of pharmacists’ intervention. According to J. Yang (2019), outpatient prescriptions for children 29 days to 12 years of age had a higher risk of dosage errors than prescriptions for teenagers and adults [29]. On the other hand, the latter DRP group accounted for a high proportion of errors in most previous studies [14,16,17]. Therefore, the inappropriate dosage was one of the essential issues in prescribing for pediatric patients. High doses increased the likelihood of ADR, while low doses reduced effectiveness and prolonged treatment duration, affecting the cost of treatment. On that basis, pharmacists intervened and reminded physicians to adjust the doses accordingly.

#### 4.2.4. Dose Timing Relative to Meals

The dose timing relative to meals rate with the highest proportion decreased from 36.1% to 32.6% after the intervention (*p* < 0.05). This group of DRPs had a lower post-intervention reduction rate than those mentioned above. According to the physicians’ feedback during the intervention as dose timing relative to meals was often less noticeable, prescriptions either lacked this information or prescribed an incorrect time for the use of the drug. For this reason, the proportion of DRPs related to the time a drug was taken did not decrease much after the intervention. Therefore, clinical pharmacists needed other interventions, such as more frequent repetition of information or warnings on prescription software, to minimize this group of DRPs.

#### 4.2.5. Drug–Drug Interaction

Our study results showed that because the rate of DRPs resulting from serious drug–drug interactions or from drug combinations to avoid in both phases was low, the reduction after the intervention was not statistically significant (*p* < 0.05). Our results were similar to previous studies: Drug–drug interactions accounted for a low rate of DRPs, less than 10.0% [15,29]. Reporting DRPs and providing drug information perhaps caused little improvement in drug–drug interaction because it was difficult for physicians to remember these data. This issue should be dealt with further, for example, by building a drug–drug interaction warning system on e-prescribing software so that physicians can consider and weigh the benefits and risks when combining two or more drugs and make decisions accordingly.

### 4.3. Risk Factors

In addition to pharmacist interventions, other factors may also affect the occurrence of DRPs. We, therefore, conducted a binary logistic regression analysis to evaluate the relationship between the survey factors (pharmacist intervention, patient age, gender, primary disease, comorbidities, number of drugs in prescription) and DRPs. Results showed that post-intervention prescriptions were less likely to lead to DRPs than pre-intervention prescriptions (OR = 0.478, 95% CI 0.436–0.524; *p* < 0.001). This analysis helped to evaluate the effectiveness of pharmacist interventions in reducing DRPs. We repeated the intervention several times in different formats to help physicians remember more information. Therefore, the intervention was effective and can significantly reduce DRPs in clinical practice, thereby helping to reduce their impact on treatment effectiveness, patient safety, and treatment costs.

In addition, the binary regression analysis results indicated no relationship between the patient’s age and the occurrence of DRPs related to the prescription (*p* > 0.05). This result was consistent with results from other studies [8]. However, associations did exist between the patient’s gender, the primary disease, the comorbidities, the number of prescription drugs, and the number of DRPs (*p* < 0.001). Prescriptions for male patients were more likely to lead to DRPs than prescriptions for females (OR = 1.124, 95% Cl 1.028–1.230; *p* = 0.011). Prescriptions for other diseases such as digestive disorders, infections, and dermatological disorders had a lower likelihood of DRPs than respiratory diseases (OR = 0.785, 95% Cl 0.712–0.866; *p* < 0.001). The number of drugs prescribed to treat respiratory diseases was often higher than for other groups of diseases. On the other hand, most drugs associated with DRPs were drugs that acted in the respiratory tract, especially in terms of inconsistent doses, which consequently increased the likelihood of DRP occurrence. Comorbidity status was also a factor related to DRPs. Patients with comorbidities were less likely to develop DRPs than patients with a single diagnosis (OR = 0.707, 95% Cl 0.627–0.798; *p* < 0.001).

Finally, the number of drugs in the prescription was also associated with DRPs. Specifically, prescriptions with ≥5 drugs were more likely to lead to DRPs than those with <5 drugs (OR = 3915, 95% Cl 3.234–4.739; *p* < 0.001). Taher Y. A (2018) also concluded that the higher the number of drugs in outpatient prescriptions, the greater the likelihood of prescription error [30]. Consequently, the higher the number of drugs, the more likely the prescription would lead to DRPs. DRPs were more frequent because each prescription might have one or more types of DRPs.

### 4.4. Study Limitations and Implementations

In this study, pharmacist intervention reduced the rate of overall DRPs and kinds of DRPs. Still, the intervention focused only on hospital-wide reporting and drug information based on identified DRPs. Pharmacists did not intervene case-by-case with each physician regarding DRPs. Therefore, after the intervention, the proportion of prescriptions associated with DRPs was still relatively high. Our study only evaluated and made interventions for eight months. In addition, it had not yet comprehensively assessed drug-related problems such as ADRs. Since different physicians often have different types of DRPs, we recommend that the following study provide drug information for each physician or specialist based on the above intervention programs. The drug information would be more concise and easier to remember, resulting in a higher intervention effect. 

Our study was conducted with a large sample size in Vietnam. We collected outpatient prescriptions from most physicians in the studied hospital. We identified DRPs based on various national and international references commonly used in clinical practices. Pharmacists repeated the intervention several times, making the physicians more alert to DRPs to improve them. The downturn in DRPs also helped increase treatment efficiency, patient safety, and cost savings. Further studies should be conducted to prove the benefits of pharmacist-led interventions on outpatient prescribing. Clinically, pharmacists might also update drug information on the hospital’s e-prescribing software and directly intervene in the software in case of DRPs, requesting physicians to change prescriptions accordingly.

## 5. Conclusions

Drug-related problems in pediatric prescriptions were quite common. The pharmacist-led interventions play a crucial role in statistically decreasing the occurrence of DRPs in prescribing, including drug selection, dosage form, dose selection, and dose time relative to meals. The incidence of DRPs in post-intervention prescriptions was less likely than in pre-intervention prescriptions among pediatric outpatients. Moreover, patient gender, primary disease, comorbidity status, and the total number of drugs prescribed were also factors associated with the occurrence of DRPs.

## Figures and Tables

**Table 1:** Contents of interventions and implementations.

Content	How to Intervene
Week 1: Reporting DRPs at a briefing meeting or medical review of the whole hospital	
1. The background of DRPs in hospital outpatient prescriptions, specific cases and consequences of DRPs; 2. A list of active ingredients for each DRP group should be kept in mind when prescribing, relevant recommendations on drug choice, dosage form, dose selection, dose timing relative to meals, and drug–drug interactions.	3. A clinical pharmacist reported DRPs with a PowerPoint presentation and presented a brief list of drugs that occurred DRPs; 4. Two other clinical pharmacists directly answered the questions and feedback of physicians during the meeting.
Week 2: Providing appropriate drug information for physicians	
The list of active ingredients for each DRPs group should be kept in mind when prescribing, relevant recommendations on drug choice, dosage form, dose selection, dose timing relative to meals, and drug–drug interactions.	5. A pharmacist emailed the hospital’s internal file to all physicians (first time) and repeated it for three days (second time); 6. Clinical pharmacists handed out printed copies to physicians (one time per physician) to receive information for all physicians who would prescribe in pre-intervention.
Week 3: Repeating the intervention contents	
The list of active ingredients for each DRP group should be kept in mind when prescribing, relevant recommendations on drug choice, dosage form, dose selection, dose timing relative to meals, and drug–drug interactions.	Clinical pharmacists talk directly or call (one time per physician) to remind physicians of the drug information provided by the pharmacist when prescribing.
In 3 weeks of intervention: Counseling prescribing	
Take note of the physicians’ responses and answer questions, provide more information, or make suggestions when required.	The physicians asked directly or phoned the pharmacists. Depending on each problem, the pharmacists answered immediately or called to answer after finding more information to provide to the physicians.

**Table 2:** Patient characteristics in pre- and post-intervention prescriptions.

Characteristics	Pre-Intervention (*n* = 4218)		Post-Intervention (*n* = 4128)		*p*-Value *
	*n*	%	*n*	%	
Age					0.164
≤2 years old	1374	32.6	1452	34.7	
>2 to ≤6 years old	1879	44.5	1830	43.8	
>6 to ≤12 years old	780	18.5	720	17.2	
>12 years old	185	4.4	180	4.3	
Gender					0.187
Female	1867	44.3	1911	45.7	
Male	2351	55.7	2271	54.3	
Primary disease (ICD-10)					<0.001
Respiratory system	3290	78.0	2431	58.1	
Other diseases	928	22.0	1751	41.9	
Comorbidity status					<0.001
No	3394	80.5	3512	84.0	
Yes	824	19.5	670	16.0	
Total drugs in prescription					<0.001
<5 drugs	3615	85.7	3945	94.3	
≥5 drugs	603	14.3	237	5.7	

* Using the χ^2^ test and the Fisher test when applicable.

**Table 3:** Drug-related problems in pre- and post-intervention prescriptions.

DRPs	Pre-Intervention(*n* = 4218)		Post-Intervention(*n* = 4128)		*p*-Value *
	*n*	%	*n*	%	
DRPs proportion					
At least one DRP	2788	66.1	1901	45.5	<0.001
1 DRP	1838	43.6	1505	36.0	<0.001
2–5 DRPs	950	22.5	396	9.5	<0.001
Average number of DRPs per prescription ± SD	0.93 ± 0.7		0.56 ± 0.7		<0.001
DRPs group proportions					
Drug choice	297	7.0	118	2.8	<0.001
Inappropriate drug for diagnosis	81	1.9	16	0.4	<0.001
Inappropriate drug for patients	216	5.1	102	2.4	<0.001
Dosage form	396	9.4	137	3.3	<0.001
Dose selection	1500	35.6	653	15.6	<0.001
Dose too high	930	22.0	372	8.9	<0.001
Dose too low	626	14.8	296	7.1	<0.001
Dose timing relative to meals	1522	36.1	1362	32.6	0.001
Major drug–drug interaction	10	0.2	4	0.1	0.112

* Using Independent-Sample *t*-test and Mann–Whitney U test when applicable.

**Table 4:** Risk factors associated with the occurrence of drug-related problems.

Characteristics	DRPs		OR		*p*-Value *
	No *n* (%)	Yes *n* (%)	(95% Confidence Intervals)		
Intervention					
No	1430 (33.9)	2788 (66.1)	0.478 (0.436–0.524)		<0.001
Yes	2281 (54.5)	1901 (45.5)			
Age					
≤2 years old	1263 (44.7)	1563 (55.3)			
>2 to ≤6 years old	1592 (42.9)	2117 (57.1)	1.041 (0.94–1.154)		0.439
>6 to ≤12 years old	856 (45.9)	1009 (54.1)	0.970 (0.858–1.096)		0.624
Gender					
Female	1737 (46)	2041 (54)	1.124 (1.028–1.230)		0.011
Male	1974 (42.7)	2648 (57.3)			
Primary disease (ICD-10)					
Respiratory system	2327 (40.7)	3394 (59.3)	0.785 (0.712–0.866)		<0.001
Other disease	1384 (51.7)	1295 (48.3)			
Comorbidity status					
No	3017 (43.7)	3889 (56.3)	0.707 (0.627–0.798)		<0.001
Yes	694 (46.5)	800 (53.5)			
Total drugs in prescription					
<5 drugs	3569 (47.2)	3991 (52.8)	3.915 (3.234–4.739)		<0.001
≥5 drugs	142 (16.9)	698 (83.1)			

* Using multivariate logistic regression model, the variable Enter method.

## Data Availability

Data sharing not applicable.
